# *Arabidopsis *eIF2α kinase GCN2 is essential for growth in stress conditions and is activated by wounding

**DOI:** 10.1186/1471-2229-8-134

**Published:** 2008-12-24

**Authors:** Sébastien Lageix, Elodie Lanet, Marie-Noëlle Pouch-Pélissier, Marie-Claude Espagnol, Christophe Robaglia, Jean-Marc Deragon, Thierry Pélissier

**Affiliations:** 1 Université de Perpignan Via Domitia, CNRS-IRD UMR5096 LGDP, 52 avenue Paul Alduy 66860 Perpignan Cedex, France; 2 Université Luminy, CNRS-CEA UMR6191 LGBP, 13009 Marseille Cedex 9, France; 3 Université Blaise Pascal, GReD UMR CNRS 6247, 24 Avenue des Landais, 63177 Aubière, France

## Abstract

**Background:**

Phosphorylation of eIF2α provides a key mechanism for down-regulating protein synthesis in response to nutrient starvation or stresses in mammalian and yeast cells. However, this process has not been well characterized in plants

**Results:**

We show here that in response to amino acid and purine starvations, UV, cold shock and wounding, the *Arabidopsis *GCN2 kinase (AtGCN2) is activated and phosphorylates eIF2α. We show that AtGCN2 is essential for plant growth in stress situations and that its activity results in a strong reduction in global protein synthesis.

**Conclusion:**

Our results suggest that a general amino acid control response is conserved between yeast and plants but that the plant enzyme evolved to fulfill a more general function as an upstream sensor and regulator of diverse stress-response pathways. The activation of AtGCN2 following wounding or exposure to methyl jasmonate, the ethylene precursor 1-Aminocyclopropane-1-carboxylic acid (ACC) and salicylic acid, further suggests that this enzyme could play a role in plant defense against insect herbivores.

## Background

Phosphorylation of the α subunit of eukaryotic translation initiation factor 2 (eIF2α) provides a key mechanism for down-regulating protein synthesis in response to nutrient starvation or stresses [1,2]. Vertebrates use four different eIF2α-kinases (PKR, PERK, HRI and GCN2) to respond to various stresses, and typically one kinase has a predominant role in response to a specific cellular stress condition. For example, in mammals, GCN2 is the primary eIF2α-kinase in response to nutrient limitation [3], PERK modulates gene expression in response to protein misfolding in the endoplasmic reticulum (ER) [4], PKR participates in an antiviral pathway mediated by interferon [5], and HRI couples protein synthesis (predominantly globin) to the availability of heme in the erythroid cell lineage [6]. However, this specialization of function is not complete and, for example, GCN2 is a secondary eIF2α-kinase in response to ER stress in mouse [7]. Arthropods make a more or less specialized use of eIF2α-kinases. The mosquito *Anopheles gambiae *has three distinct enzymes (HRI, PERK and GCN2) while *Drosophila melanogaster *and *Caenorhabditis elegans *have only two (PERK and GCN2) [8]. The situation is similar for fungi since *Schizosaccharomyces pombe *has three enzymes (two distinct HRI and a GCN2) while *Saccharomyces cerevisiae *and *Neurospora crassa *have a single eIF2α-kinase (GCN2) [8].

In amino acid starved *S. cerevisiae *cells, GCN2-dependent eIF2α phosphorylation leads to a general down regulation of protein synthesis and to the increased translation of the GCN4 mRNA [2]. Following translation, the GCN4 transcriptional activator can induce the transcription of more than 500 genes, including the majority involved in amino acid synthesis. Uncharged tRNAs that accumulate during amino acid starvation activate GCN2 by binding to a histidyl-tRNA synthetase-related domain located C-terminal to the kinase domain [2]. *S. cerevisiae *GCN2 is activated not only by nutrient limitation including amino acid, purine and glucose deprivation, but also by high concentration of sodium and by the immunosuppressant rapamycin [2].

In plants, the lower dissociation constant of eIF2 for GDP compared to yeast or mammals have led several years ago to the assumption that plants do not use phosphorylation of eIF2α as a mean to regulate translation [9]. Furthermore, the observation that starvation for aromatic or branched amino acids did not initiate a general response have also led to the suggestion that, in contrast to yeast, such general amino acid response may not exist in plants [10]. Therefore the physiological significance of eIF2α phosphorylation in plants is largely unknown. More recently, a GCN2-like enzyme (AtGCN2) has been identified in *Arabidopsis thaliana *and was shown to restore the growth of a yeast *gcn2 *mutant in the presence of an inhibitor of amino acids biosynthesis [11]. AtGCN2 was also shown to be activated in plants by amino acid deprivation conditions [12].

In this work, we show that in response to several stresses, including amino acid and purine starvations, UV, cold shock and wounding, *Arabidopsis *GCN2 kinase is activated and phosphorylates eIF2α. We show that GCN2 is essential for plant growth in stress situations and that this activity is linked to a strong reduction in global protein synthesis. The activation of GCN2 following wounding or exposure to methyl jasmonate, ACC and salicylic acid, further suggests that this enzyme could play a role in plant defense responses to insect herbivores and may represent a key but yet uncharacterized player linking biotic and abiotic stresses.

## Results and discussions

### *Arabidopsis *and rice have a single eIF2α kinase

In addition to the presence of a GCN2 enzyme, higher plants have been proposed to contain a double-stranded RNA-dependent protein kinase with biochemical properties of the mammalian PKR [13-15]. Using the protein kinase domain of the yeast and *Arabidopsis *GCN2 and of the human PKR, PERK and HRI enzymes, we searched the *Arabidopsis *genome for the presence of additional eIF2α kinases. GCN2, PRK, PERK and HRI protein kinase domains were used in a BLASTP analysis against the TAIR 8.0 *Arabidopsis *proteins  and, in each case, fifty proteins with the highest E value were collected. Using this approach, we produced a list of 138 non-redundant kinases (out of the more than 1000 *Arabidopsis *kinases) that most closely resemble eIF2α kinases (Additional file 1). Using a phylogenetic approach, we compared these 138 kinase domains to yeast, human and mouse eIF2α kinase domains. Two *Arabidopsis *kinases were found to form a statistically significant cluster with known eIF2α kinases (Additional file 1): one is AtGCN2 (AT3G59410) while the other is annotated as a Wee1-like kinase (AT1G02970) [16]. To evaluate the capacity of the Wee1-like protein to bind to eIF2α, we analyzed in more detail the alignment of this enzyme to the other eIF2α kinases (Additional file 2). We observed that three essential structural characteristics of eIF2α kinases are not present in the Wee1-like kinase: a large insert (more than 30 amino acids) between the conserved kinase domains IV and V [17], a threonine in a putative autophosphorylation site (position 446 for the human PKR) [18] and a threonine at the end of domain IX (position 487 for the human PKR) that is critical for the substrate specificity [18]. Therefore, although the *Arabidopsis *Wee1-like enzyme possess a high degree of homology to eIF2α kinases, as previously noted for the human Wee1 [19], it is unlikely to bind to eIF2α. We conclude that *Arabidopsis *likely possesses a single eIF2α kinase of the GCN2 type. The same conclusion was obtained using this approach to analyze the rice genome (data not shown) suggesting that higher plants only contain a GCN2-like eIF2α kinase.

### *Arabidopsis *thaliana GCN2 can phosphorylate eIF2α and is essential in amino acid deprivation conditions

**Figure 1:**
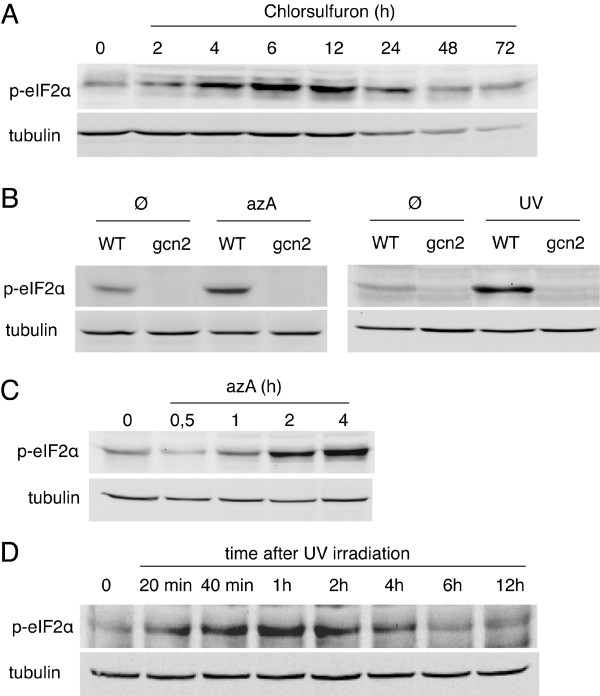
**AtGCN2 phosphorylates eIF2α during amino-acid and purine deprivation or after UV irradiation**. A. Time-course of eIF2α phosphorylation in response to amino acid starvation induced by chlorsulfuron. B. Immunoblot analysis of eIF2α phosphorylation in wild-type (WT) or *gcn2 *mutant seedlings, untreated (Ø), starved for purine with 8-azaadenine (azA) for 4 h or irradiated with UV. C. Time-course of eIF2α phosphorylation in response to purine starvation induced by azA. D. Time-course of eIF2α phosphorylation in response to UV irradiation. Phosphorylation of eIF2α was measured by using an antibody that specifically recognizes the phosphorylated form (upper panel). Levels of total protein were assayed by using an antibody that recognizes tubulin (lower panel).

**Figure 2:**
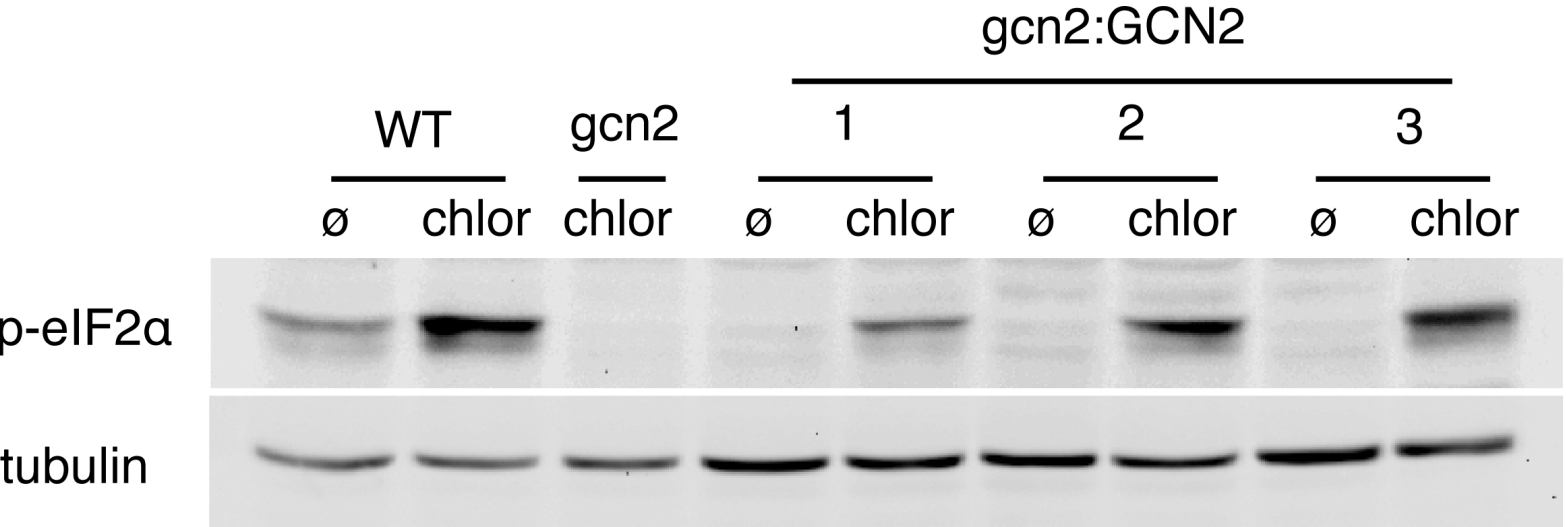
**Molecular complementation of the *gcn2 *mutant line by the expression of AtGCN2**. Immunoblot analysis of eIF2α phosphorylation in wild type (WT), *gcn2 *mutant or in three independent *gcn2 *transgenic lines expressing an intact AtGCN2 (gcn2:GCN2). Seedlings were either untreated (Ø) or starved for branched amino acids with chlorsulfuron (chlor) for 6 hours.

To investigate the role of AtGCN2, we first decided to confirm whether amino acid depletion, a stress known to activate yeast GCN2, is actually inducing eIF2α phosphorylation in *Arabidopsis*. Similarly to the results recently reported by Zhang et al. [12], we observed that aromatic or branched amino acid deprivation (a situation generated by exposing plants to glyphosate or chlorsulfuron respectively [20]) both induce eIF2α phosphorylation, an induction that is completely suppressed by adding the appropriate amino acids to the growth medium (data not shown). eIF2α phosphorylation occurs only 4 hours after disrupting the branched amino acid synthesis pathway and peaks after 6 hours of treatment (Figure 1A). eIF2α phosphorylation is completely dependent on the presence of GCN2. Indeed, no phosphorylation was detected in a GCN2 null mutant line after branched and aromatic amino acid deprivation (Figure 2 and not shown ; see Additional file 3 for a description of the *gcn2 *mutant line).

In a classical rich medium, *gcn2 *mutant plants do not have a particular phenotype compared to wild-type plants. However, we observed that *gcn2 *plants are much more sensitive to chlorsulfuron herbicide treatment than wild-type plants (data not shown). Following 6 days of chlorsulfuron-induced amino acid starvation *gcn2 *seedlings appeared smaller and more chlorotic than wild-type seedlings, a result that concurs with that of Zhang et al. [12]. The introduction of an intact copy of AtGCN2 in the *gcn2 *mutant line allows the plants to grow identically to wild-type seedlings during amino acid deprivation (data not shown), confirming that AtGCN2 is essential for plant growth in this stress situation. As expected, rescue of eIF2α phosphorylation in response to amino acid deprivation was also observed in the complemented *gcn2*:GCN2 plants (Figure 2). These results confirm that AtGCN2 is the only *Arabidopsis *kinase involved in eIF2α phosphorylation in amino acid starvation conditions.

### Characterization of other stresses leading to eIF2α phosphorylation in *Arabidopsis*

**Figure 3:**
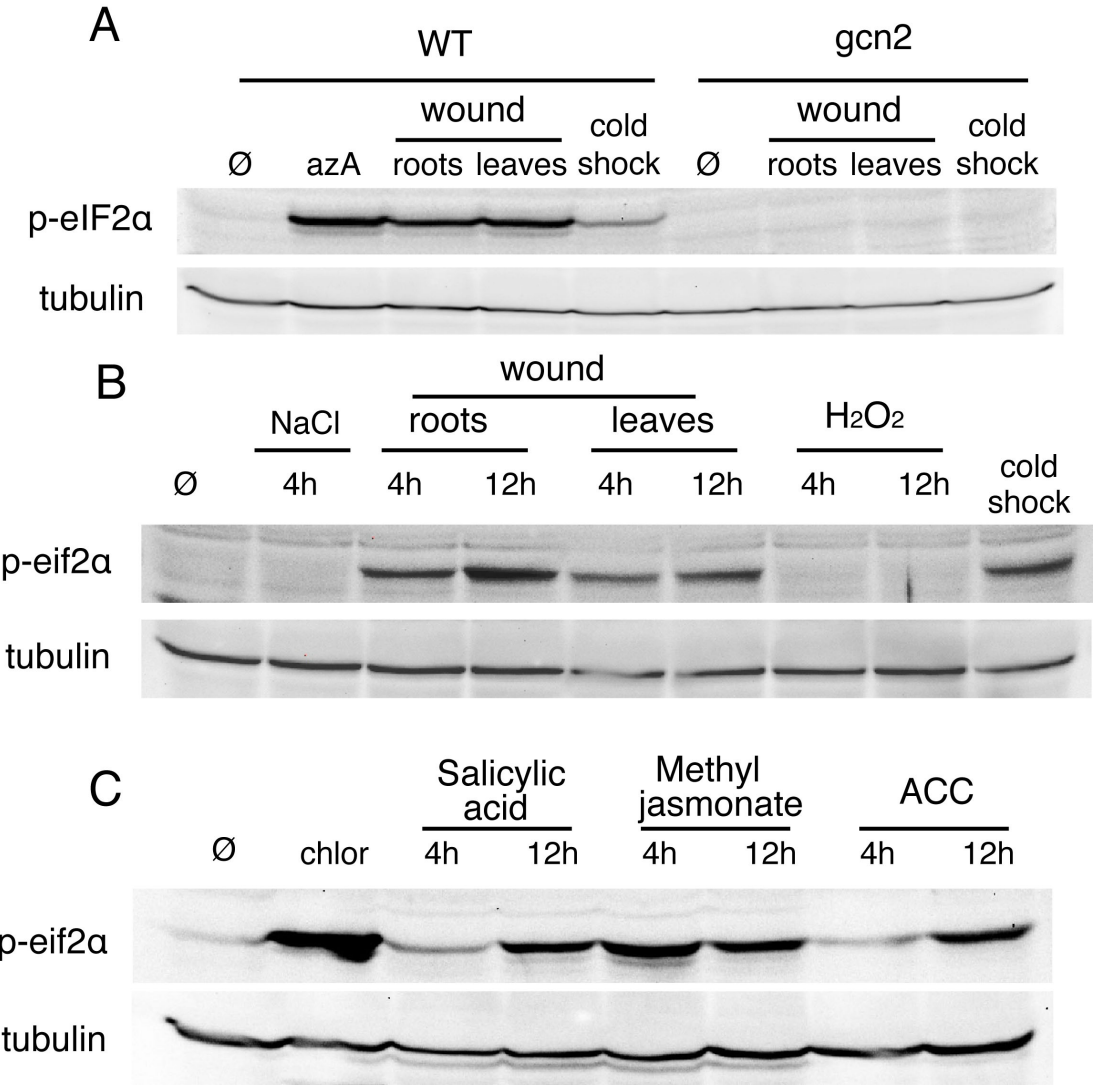
**Stress conditions leading to GCN2-dependent eIF2α phosphorylation in *Arabidopsis***. A. Immunoblot analysis of eIF2α phosphorylation in wild type (WT) or *gcn2 *mutated seedlings. Seedlings were either left untreated (Ø), starved for purine with azA, exposed to low temperature (cold shock) or wounded on their leaves or roots. B. eIF2α phosphorylation in wild type untreated (Ø) seedlings, or seedlings exposed to NaCl, H_2_O_2_, low temperature (cold shock), or wounded on their leaves or roots (wound) C. eIF2α phosphorylation in wild type seedlings, untreated (Ø) or exposed to chlorsulfuron, salicylic acid, methyl jasmonate or 1-aminocyclopropane-1-carboxylic acid (ACC). In addition, for methyl jasmonate, eIF2α phosphorylation was already detected after 30 minutes of induction (Additional file 4).

Purine deprivation is known to activate yeast GCN2 [2]. We observed that purine deprivation (a situation generated by exposing plants to 8-azaadenine (azA) [21]) is also inducing eIF2α phosphorylation in *Arabidopsis *(Figure 1B). Interestingly, this phosphorylation appears within 2 hours after purine limitation (Figure 1C) and likely results of a reduction in tRNA aminoacylation efficiency leading to the accumulation of uncharged tRNAs [2]. eIF2α is also quickly phosphorylated after UV irradiation (Figure 1B). This response begins only 20 minutes after irradiation, with a maximum of phosphorylation after 1 hour and a return to basal levels after 6 hours (Figure 1D). Again, both phosphorylation events are completely dependent on the presence of GCN2 as no eIF2α phosphorylation could be detected in the mutant *gcn2 *line in stress condition (Figure 1B). Although oxidative and osmotic stresses are known to activate yeast GCN2 [2,22], we were not able to activate AtGCN2 using NaCl or H_2_O_2 _(see Figure 3B). Also, heat shock did not lead to eIF2α phosphorylation in *Arabidopsis *(data not shown) confirming previous results obtained in wheat [23]. In contrast, cold shock, plant wounding, methyl jasmonate, 1-aminocyclopropane-1-carboxylic acid (ACC) and salicylic acids were efficient, plant-specific inducer of GCN2-dependent eIF2α phosphorylation (Figure 3 and Additional file 4). Jasmonate acid (JA) and related signaling compounds such as methyl jasmonate are ubiquitous signals for tissue injury and for the subsequent activation of defense response to insect herbivores [24]. ACC is a precursor of ethylene, which also affects the expression of defensive proteins and secondary metabolites in response to insect herbivores [24]. Both ACC and JA accumulate simultaneously in response to insect herbivores and form a conjugate (JA-ACC) that was proposed to coordinately regulate the plant defense response [24]. Finally, salicylic acids is well known to regulate the plant defense response to lepidopteran insects [24]. The common capacity of these three key hormones to induce eIF2α phosphorylation suggests that AtGCN2 participates in the plant defense response to insect herbivores.

### The AtGCN2 activity is not regulated by the TOR pathway

**Figure 4:**
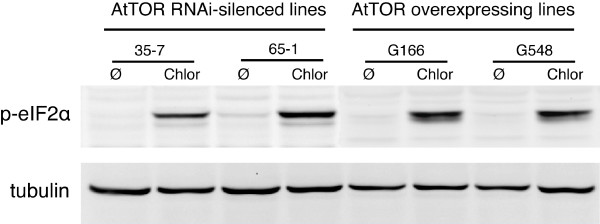
**The TOR pathway does not interfere with AtGCN2 activity**. Immunoblot analysis of eIF2α phosphorylation in TOR RNAi-silenced (35-7 and 65-1) or TOR-overexpressing (G166 and G548) lines (described in [25]). Seedlings of the different backgrounds were either left untreated (Ø) or starved for branched amino acids using chlorsulfuron (Chlor) for 4 h.

TOR (target of rapamycin) protein kinase is a central regulator of cell growth in response to nutrient and growth factors [21]. A TOR homolog has been identified in *Arabidopsis *(AtTOR) and has been implicated in embryo development, meristem-driven cell growth, osmotic stress inhibition and the stimulation of mRNA translation [25]. In yeast, phosphorylation of eIF2α by GCN2 is responsible for about 50% of the inhibition of translation initiation following the use of the TOR inhibitor rapamycin, suggesting that an important cross-talk exist between TOR and GCN2 pathways [21]. In *Arabidopsis*, the RNAi inhibition of the TOR pathway also results in a strong inhibition of translation initiation while TOR overexpressing lines present an increase in translation initiation efficiency [25]. To evaluate the implication of AtGCN2 in this regulation, we analyzed the level of eIF2α phosphorylation in AtTOR RNAi-silenced (35-7, 65-1) and overexpressing (G548, G166) lines in normal and amino acid deprivation conditions (Figure 4). The partly silenced 35-7 and 65-1 lines show a significant decrease in the accumulation of AtTOR mRNA and a severe reduction in shoot and root growth while the two overexpressing G548 and G166 lines have opposite features (increase accumulation of AtTOR mRNA and a strong increase in shoot and root growth) [25]. No increase in eIF2α phosphorylation was detected in unstressed TOR-silenced lines and a similar pattern of phosphorylation was observed in stressed TOR-silenced and overexpressing lines (Figure 4) suggesting that, in contrast to the yeast situation, no cross-talk exists between the TOR and GCN2 pathways in *Arabidopsis*.

### GCN2 activity induces a strong reduction in global protein synthesis

**Figure 5:**
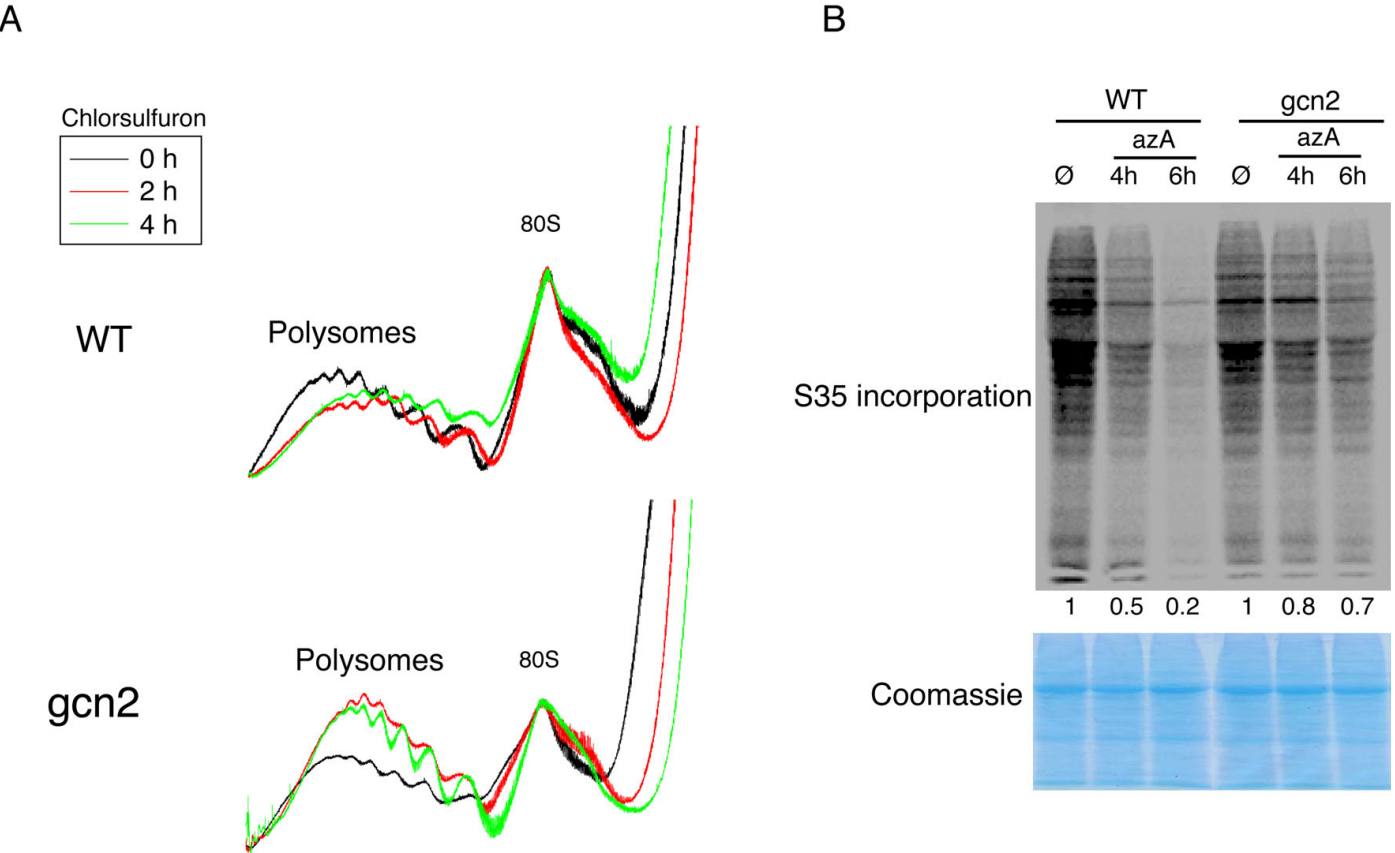
**AtGCN2 is a regulator of general protein synthesis during amino acids and purine starvation**. A. Absorbance profile at 254 nm of ribosomes purified by ultracentrifugation on a sucrose density gradient. The ribosomal pellet fraction was prepared from wild type (WT) or *gcn2 *mutant seedlings starved for amino acid using chlorsulfuron for 2 or 4 hours. The positions of the monosomes (80S) and polysomes are indicated. B. 35S-labeled methionine incorporation of wild-type (WT) or *gcn2 *mutant seedlings starved for purine using azA for 4 to 6 hours. A fraction of the total protein extract was separated on a SDS-polyacrylamide gel, desiccated and exposed to a phosphoimager screen for quantification (upper panel). For each genotype, the relative amount of labeled proteins is indicated under each lane after normalization against the untreated sample set arbitrarily to 1. An equal fraction of the total protein extract was stain with coomassie blue as a loading control (lower panel).

The impact of eIF2α phosphorylation on the efficiency of mRNA translation was next evaluated. We observed a reduction in the abundance of high-molecular weight polysomes compared to monosomes in amino acids (Figure 5A) or purine (Additional file 5) deprived wild type plants. This variation in the polysomes to monosomes ratio is diagnostic of a reduction in translation elongation rate and translation initiation efficiency caused by these stress conditions. In contrast, we found that amino acids (Figure 5A) or purine (Additional file 5) deprived *gcn2 *mutant overaccumulates high molecular weight polysomes compared to monosomes. This suggests that, in these stress conditions, *gcn2 *mutant can no longer prevent translation initiation in a context where translation elongation is slowed down. In support to this conclusion, we observed a marked decrease in the amount of neo-synthesized proteins by wild type plant during purine deprivation (Figure 5B), a decrease that is much weaker in *gcn2 *mutant plants (Figure 5B). Globally, these results suggest that the GCN2-dependent increase in eIF2α phosphorylation under amino acids or purine deprivation conditions, results in a strong reduction of protein synthesis.

## Conclusion

Our data indicate that AtGCN2 is important for plant growth in stress situations, likely through the general down regulation of mRNA translation. For the moment, it is not known if eIF2α phosphorylation by AtGCN2 can lead to the selective stimulation of specific mRNA translation like this is the case for the yeast GCN4 [2] or mammalian ATF4 mRNAs [1]. The strong activation of AtGCN2 following wounding and exposure to key hormones suggests that this enzyme evolved to play a role in plant defense responses to insect pathogens and may represent a key but yet uncharacterized player linking biotic and abiotic stresses. Therefore, while mammalians use four different eIF2α kinases to respond to a large variety of biotic and abiotic stresses, plants apparently achieve a similar result with a single, GCN2-like enzyme.

## Methods

### Plant material, growth condition and stress treatments

Wild-type and *gcn2 *mutated *Arabidopsis thaliana *plants are from the Landsberg (ler-0) ecotype. The *gcn2 *insertion line was obtained from RIKEN Genomic Science Center . Sterile seeds were germinated on solid Murashige and Skoog (MS) medium containing 1% sucrose (w/v) and cultured in a 16-h-light/8-h-dark cycle for 10 days at 23°C. When appropriate, Chlorsulfuron, glyphosate, 8-azaadenine, methyl jasmonate, salicylic acid, ACC and branched amino-acid were added to the medium at a final concentration of 0.6 μM, 1.5 mM, 50 μg/ml, 25 μM, 0.6 mM, 50 μM and 5 mM respectively. For the UV stress, seedlings were irradiated with UV light at 50 mJ/m2 by using a UV cross-linker (Bio-Rad Laboratories, Inc.). Plant wounding was performed by crushing the leaf across the midrib with a haemostat. For the cold shock treatment, seedlings were transferred at 4°C during 2 h followed by 2 h back in the growth chamber at 23°C. Heat shock were performed at 42°C during 2 h followed by 2 h back in the growth chamber at 23°C. For osmotic and oxidative stresses, the seedlings were placed in solutions containing 250 mM NaCl or 1 mM H_2_O_2 _during the time indicated.

### Immunoblot analysis of eIF2α phosphorylation

Western blots were probed using a phosphospecific anti-eIF2α rabbit monoclonal antibody (Epitomics, Burlingame, CA; 1/1,000 dilution). After incubation with a horseradish peroxydase-coupled anti-rabbit secondary antibody (Sigma 1/5,000 dilution) immunoblots were developed by using the ECL Plus Western Blotting detection reagents (GE Healthcare Bio-Sciences). Chemiluminescence was visualized with a VersaDoc Imaging System (Bio-Rad Laboratories, Inc.). Equal loading of protein were confirmed by reprobing the membranes with a mouse monoclonal anti-alpha-tubulin (Sigma 1/5,000 dilution).

### Polysome preparation and detection of neo-synthesized proteins in normal and stress situation

For polysome preparation, seeds were sown in liquid medium, incubated 48 h at 4°C, and grown under constant light during 10 days at 23°C. Chlorsulfuron was added, and after 2 or 4 hours, the ribosomal pellet fraction was prepared from wild-type (WT) or *gcn2 *mutant seedlings as described in [25]. Polysome profiles were displayed on sucrose gradients and profiles recorded at 260 nm. For the detection of neo-synthesized proteins, the 10 days-old seedlings were treated for 2 to 4 hours with 8-azaadenine and then transferred for 2 hours into 1 ml of MS medium containing 50 μCi of ^35^S-labelled methionine and 8-azaadenine. Untreated controls were incubated for 2 hours into the same labelling medium, but in the absence of 8-azaadenine. After two washes with MS medium, total proteins were extracted as described below and 35 μg of protein were separated on SDS-PAGE. Gels were stained with Coomassie blue or dried and autoradiographied using a PhosphoImager (Bio-Rad Laboratories, Inc.).

### Preparation of total protein extracts

Following exposure to stress, total protein extracts were prepared as following. For each treatment, three seedlings were ground in Laemmli buffer containing both Complete protease and PhosSTOP phosphatase inhibitor (Roche Diagnostics, Indianapolis, IN) and incubated at 95°C for 5 min. After centrifugation, samples were fractioned by sodium dodecyl sulphate-polyacrylamide gel electrophoresis (SDS-PAGE) and transferred to nitrocellulose.

## Authors' contributions

SL carried out the molecular genetic studies on GCN2, participated in the polysome profile analysis and drafted the manuscript. EL did the polysome profile analysis and, with CR, helped in its interpretation. MNP and MCE provided technical help on the molecular genetic work. JMD and TP conceived the study, participated in its coordination and final writing of the manuscript. All authors read and approved the final manuscript.

## Supplementary Material

Additional file 1 **Evolutionary tree of the kinase domains of 138 *Arabidopsis *enzymes most closely related to GCN2, PKR, PERK and HRI eIF2α kinases.** Click here for file

Additional file 2 **Multiple sequence alignment of the kinase domains of the eIF2α kinase group on the evolutionary tree of Additional file **1**, performed using MUSCLE (v3.7).** Click here for file

Additional file 3 **Description of the *gcn2 *mutant line.** Click here for file

Additional file 4 **Phosphorylation of eIF2α in response to hormones.** Click here for file

Additional file 5 **Absorbance profile at 254 nm of ribosomes purified by ultracentrifugation on a sucrose density gradient.** Click here for file
