# Supplementary figures and images for: Arabidopsis eIF2α kinase GCN2 is essential for growth in stress conditions and is activated by wounding
Source: BMC Plant Biol. 2008 Dec 24;8:134. doi: 10.1186/1471-2229-8-134 (PMC2639386; doi:10.1186/1471-2229-8-134)

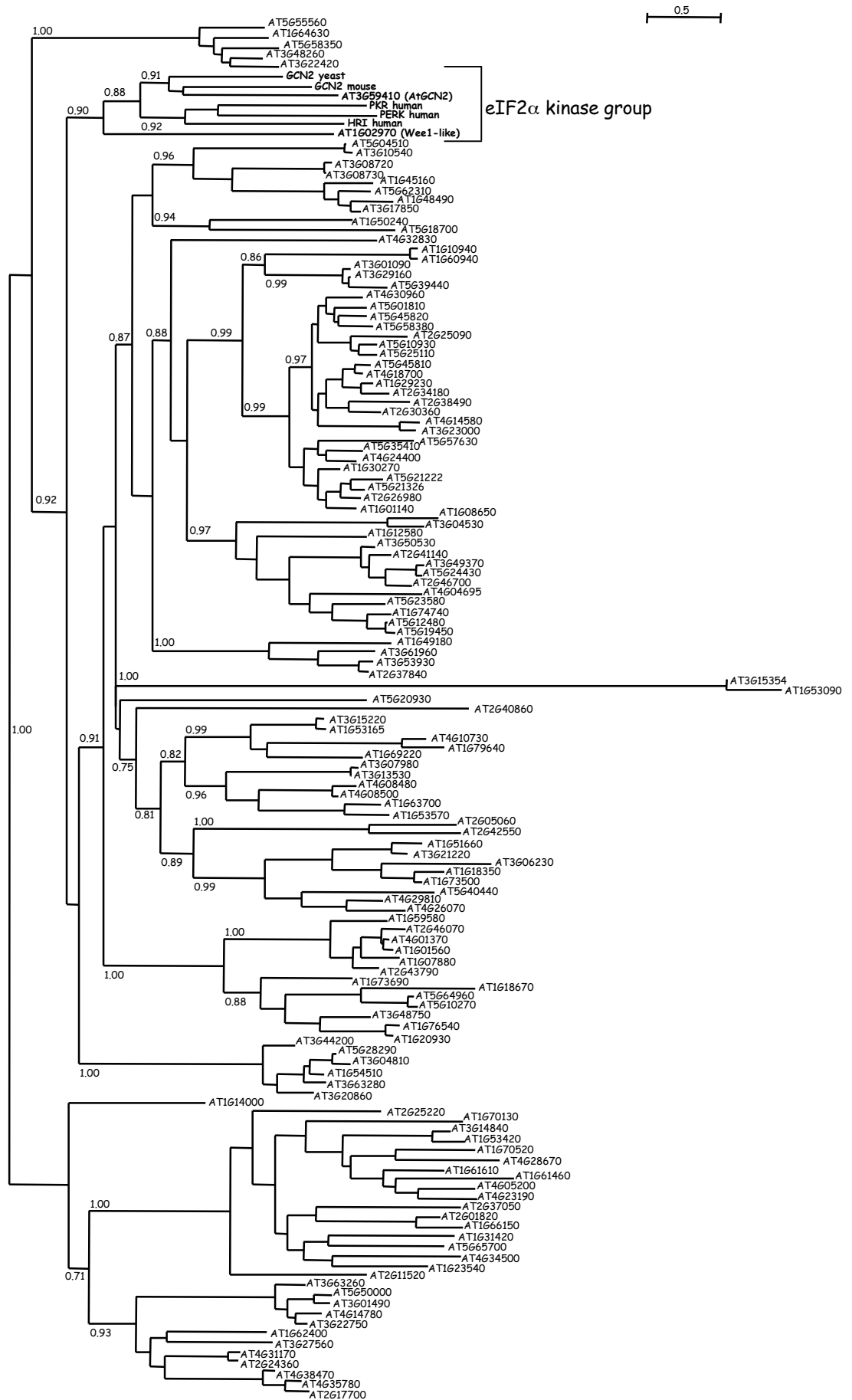

Supplement: Additional file 1 — Evolutionary tree of the kinase domains of 138 Arabidopsis enzymes most closely related to GCN2, PKR, PERK and HRI eIF2α kinases. [file 1471-2229-8-134-S1.pdf]

A

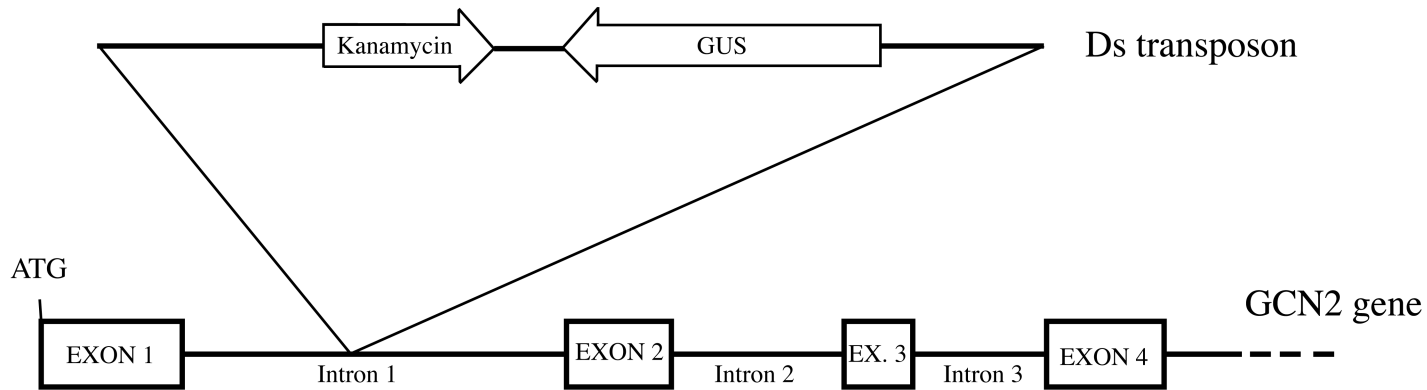

B

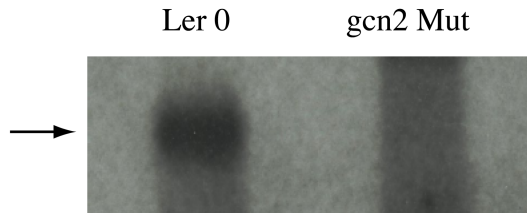

Supplement: Additional file 3 — Description of the gcn2 mutant line. [file 1471-2229-8-134-S3.pdf]

AzA

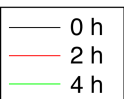

WT

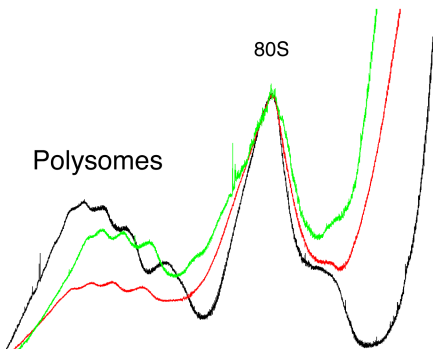

Polysomes

80S

*gcn2*

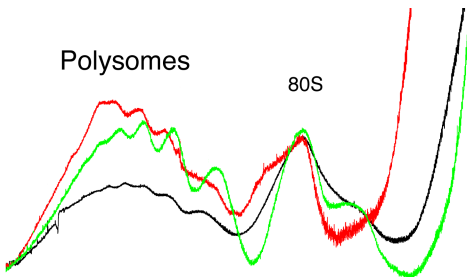

Supplement: Additional file 5 — Absorbance profile at 254 nm of ribosomes purified by ultracentrifugation on a sucrose density gradient. [file 1471-2229-8-134-S5.pdf]
