# Supplementary material for: Arabidopsis eIF2α kinase GCN2 is essential for growth in stress conditions and is activated by wounding
Source: BMC Plant Biol. 2008 Dec 24;8:134. doi: 10.1186/1471-2229-8-134 (PMC2639386; doi:10.1186/1471-2229-8-134)
Supplement: Additional file 2 — Multiple sequence alignment of the kinase domains of the eIF2α kinase group on the evolutionary tree of Additional file 1, performed using MUSCLE (v3.7). [file 1471-2229-8-134-S2.pdf]

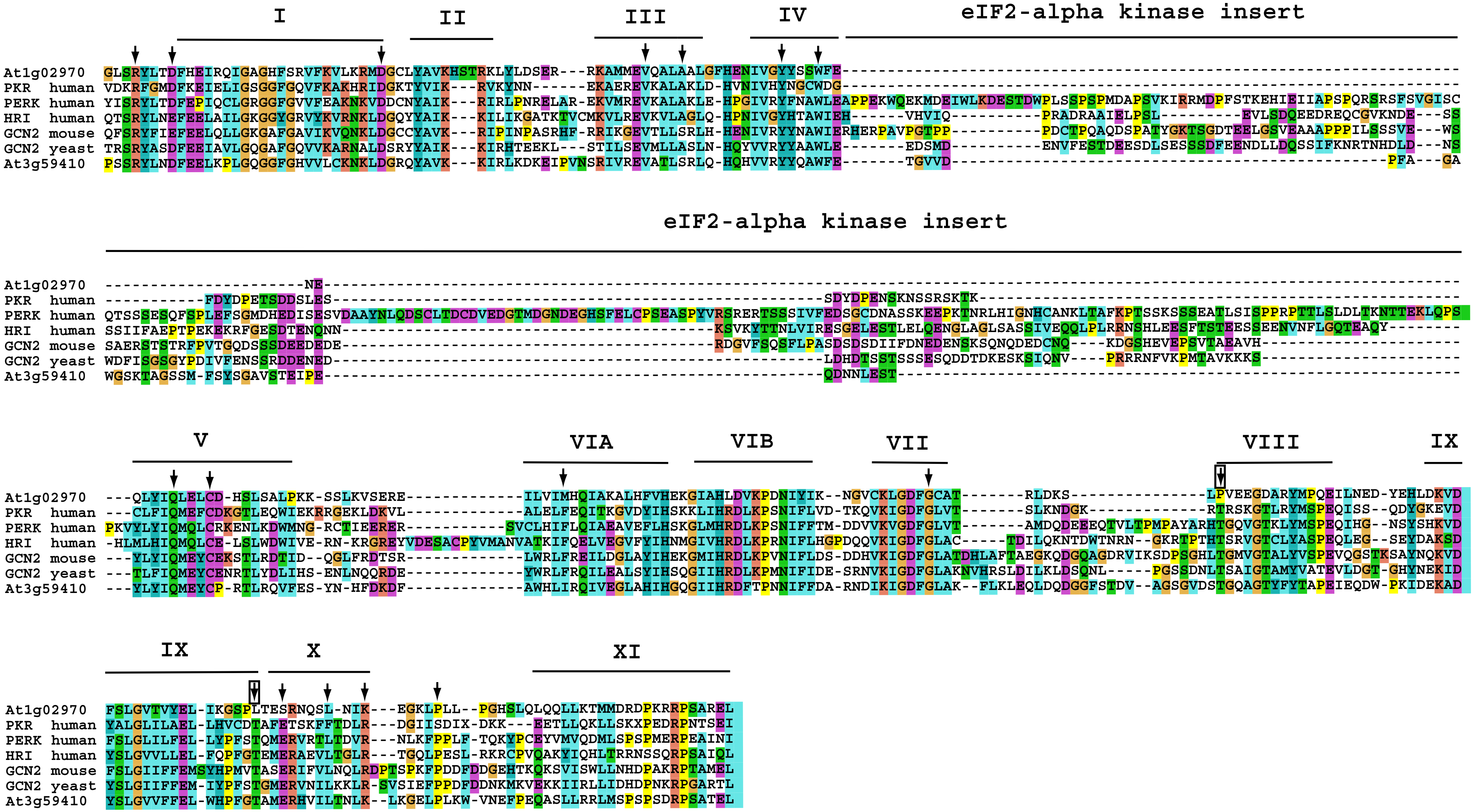
I
II
III
IV
eIF2-alpha kinase insert
At1g02970 GLSRYLTDFHEIRQIGAGHFSRVFKVLKRMDGCLYAVKHSTRKLYLDSER---RKAMMEVQALAALGFHENIVGYYSSWFE
PKR human VDKRFGMDFKEIELIGSGGFGQVFKAKHRIDGKTYVIK---RVKYNN------EKAEREVKALAKLD-HVNIVHYNGCWDG
PERK human YISRYLTDFEPIQCLGRGGFGVVFEAKNKVDDCNYAIK---RIRLPNRELAR-EKVMREVKALAKLE-HPGIVRYFNAWLEAPPEKWQEKMDEIWLKDESTDWPLSSPSPMDAPSVKIRRMDPFSTKEHIEIIAPSPQRSRSFSVGISC
HRI human QTSRYLNEFEELAILGKGGYGRVYKVRNKLDGQYYAIK---KILIKGATKTVCMKVLREVKVLAGLQ-HPNIVGYHTAWIEH------VHVIQ----------PRADRAAIELPSL---------EVLSDQEEDREQCGVKNDE---SS
GCN2 mouse QFSRYFIEFEELQLLGKGAFGAVIKVQNKLDGCCYAVK---RIPINPASRHF-RRIKGEVTLLSRLH-HENIVRYYNAWIERHERPAVPGTPP----------PDCTPQAQDSPATYGKTSGDTEELGSVEAAAPPPILSSSVE---WS
GCN2 yeast TRSRYASDFEEIAVLGQGAFGQVVKARNALDSRYYAIK---KIRHTEEKL---STILSEVMLLASLN-HQYVVRYYAAWLE-------EDSMD----------ENVFESTDEESDLSESSSDFEENDLLDQSSIFKNRTNHDLD---NS
At3g59410 PSSRYLNDFEELKPLGQGGFGHVVLCKNKLDGRQYAVK---KIRLKDKEIPVNSRIVREVATLSRLQ-HQHVVRYYQAWFE-------TGVVD---...---PFA---GA
eIF2-alpha kinase insert
At1g02970 ---------------------NE---...
PKR human -----------FDYDPETSDDSLES---...---SDYDPENSKNSSRSKTK---
PERK human QTSSSESQFSPLEFSGMDHEDISESVDAAYNLQDSCLTDCDVEDGTMDGNDEGHSFELCPSEASPYVRSRERTSSSIVFEDSGCDNASSKEEPKTNRLHIGNHCANKLTAFKPTSSKSSSEATLSIPPRPTTLSLDLTKNTTEKLQPS
HRI human SSIIFAEPTPEKEKRFGESDTENQNN---...---KSVKYTTNLVIRESGELESTLELQENGLAGLSASSIVEQQLPLRRNSHLEESFTSTEESSEENVNFLGQTEAQY--------
GCN2 mouse SAERSTSTRFPVTGQDSSDEEDEDE---...---RDGVFSQSFLPASDSDSDIIFDNEDENSKSQNQDEDCNQ----KDGSHEVEPSVTAEAVH---
GCN2 yeast WDFISGSGYPDIVFENSSRDDENED---...---LDHDTSSTSSSESQDDTDKESKSIQNV----PRRRNFVKPMTAVKKKS---
At3g59410 WGSKTAGSSM-FSYSGAVSTEIPE---...---QDNNLEST---
V
VIA
VIB
VII
VIII
IX
At1g02970 ---QLYIQLELCD-HSLSALPKK-SSLKVSERE------------ILVIMHQIAKALHFVHEKGIAHLDVKPDNIYIK--NGVCKLGDFGCAT-------RLDKS----------------LPVEEGDARYMPQEILNED-YEHLDKVD
PKR human ---CLFIQMEFCDKGTLEQWIEKRRGEKLDKVL------------ALELFEQITKGVDYIHSKKLIHRDLKPSNIFLVD-TKQVKIGDFGLVT-------SLKNDGK-------------RTRSKGTLRYMSPEQISS--QDYGKEVD
PERK human PKVYLYIQMQLCRKENLKDWMNG-RCTIEERER-----------SVCLHIFLQIAEAVEFLHSKGLMHRDLKPSNIFFTM-DDVVKVGDFGLVT-------AMDQDEEEQTVLTPMPAYARHTGQVGTKLYMSPEQIHG--NSYSHKVD
HRI human -HLMLHIQMQLCE-LSLWDWIVE-RN-KRGREYVDESACPYVMANVATKIFQELVEGVFYIHNMGIVHRDLKPRNIFLHGPDQQVKIGDFGLAC----TDILQKNTDWTNRN--GKRTPTHTSRVGTCLYASPEQLEG--SEYDAKSD
GCN2 mouse ---YLYIQMEYCEKSTLRDTID--QGLFRDTSR------------LWRLFREILDGLAYIHEKGMIHRDLKPVNIFLDS-DDHVKIGDFGLATDHLAFTAEGKQDGQAGDRVIKSDPSGHLTGMVGTALYVSPEVQGSTKSAYNQKVD
GCN2 yeast ---TLFIQMEYCENRTLYDLIHS-ENLNQQRDE------------YWRLFRQILEALSYIHSQGIIHRDLKPMNIFIDE-SRNVKIGDFGLAKNVHRSLDILKLDSQNL-----PGSSDNLTSAIGTAMYVATEVLDGT-GHYNEKID
At3g59410 ---YLYIQMEYCP-RTLRQVFES-YN-HFDKDF------------AWHLIRQIVEGLAHIHGQGIIHRDFTPNNIFFDA-RNDIKIGDFGLAK--FLKLEQLDQDGGFSTDV--AGSGVDSTGQAGTYFYTAPEIEQDW-PKIDEKAD
IX
X
XI
At1g02970 FSLGVTVYEL-IKGSPLTESRNQSL-NIK---EGKLPLL--PGHSLQLQQLLKTMMDRDPKRRPSAREL
PKR human YALGLILAEL-LHVCDTAFETSKFFTDLR---DGIISDIX-DKK---EETLLQKLLSKXPEDRPNTSEI
PERK human FSLGLILFEL-LYPFSTQMERVRTLTDVR---NLKFPPLF-TQKYPCEYVMVQDMLSPSPMERPEAINI
HRI human YSLGVVLLEL-FQPFGTEMERAEVLTGLR---TGQLPESL-RKRCPVQAKYIQHLTRRNSSQRPSAIQL
GCN2 mouse FSLGIIFFEMSYHPMVTASERIFVLNQLRDPTSPKFPDDFDDGEHTKQKSVISWLLNHDPAKRPTAMEL
GCN2 yeast YSLGIIFFEM-IYPFSTGMERVNILKKLR-SVSIEFPPDFDDNKMKVEKKIIRLLIDHDPNKRPGARTL
At3g59410 YSLGVVFFEL-WHPFGTAMERHVILTNLK--LKGELPLKW-VNEFPEQASLLRRLMSPSPSDRPSATEL
