# Supplementary material for: Arabidopsis eIF2α kinase GCN2 is essential for growth in stress conditions and is activated by wounding
Source: BMC Plant Biol. 2008 Dec 24;8:134. doi: 10.1186/1471-2229-8-134 (PMC2639386; doi:10.1186/1471-2229-8-134)
Supplement: Additional file 4 — Phosphorylation of eIF2α in response to hormones. [file 1471-2229-8-134-S4.pdf]

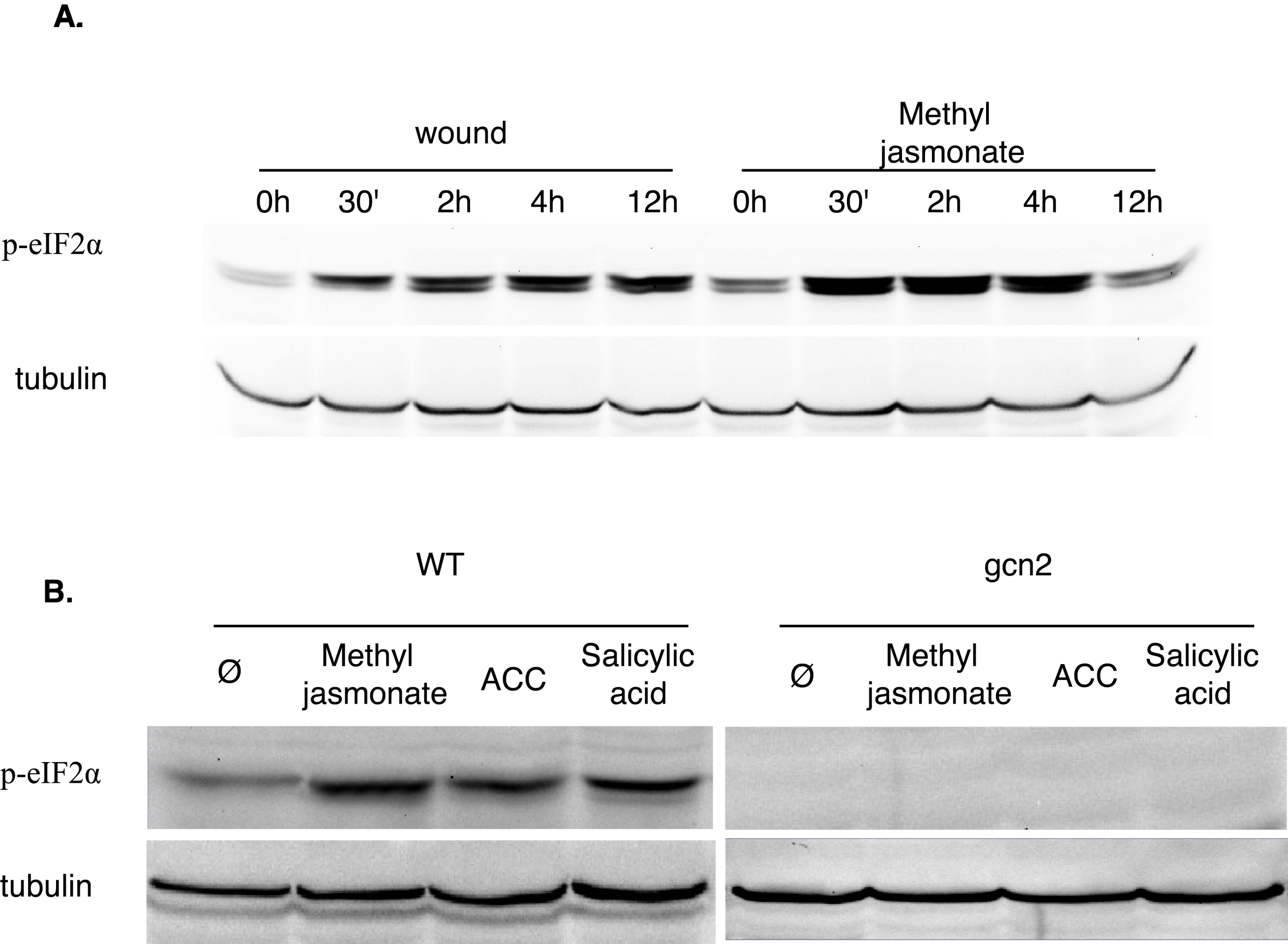
A.
wound
Methyl jasmonate
0h
30'
2h
4h
12h
0h
30'
2h
4h
12h
p-eIF2α
tubulin
B.
WT
gcn2
Ø
Methyl jasmonate
ACC
Salicylic acid
Ø
Methyl jasmonate
ACC
Salicylic acid
p-eIF2α
tubulin
